# ZZ-YOLOv11: A Lightweight Vehicle Detection Model Based on Improved YOLOv11

**DOI:** 10.3390/s25113399

**Published:** 2025-05-28

**Authors:** Zhe Zhang, Zhongyang Zhang, Gang Li, Chenxi Xia

**Affiliations:** School of Automobile and Traffic Engineering, Liaoning University of Technology, Jinzhou 121001, China; 231286015@stu.lnut.edu.cn (Z.Z.); qcxy-zzy@lnut.edu.cn (Z.Z.); xcx3113@163.com (C.X.)

**Keywords:** deep learning, vehicle inspections, lightweight, model pruning, model distillation

## Abstract

**Highlights:**

**What are the main findings?**

- Developed ZZ-YOLO with GEIT module and LDCD detection head, achieving 70.9% detection accuracy and 58% mAP@0.5 on KITTI and BDD100K datasets, improving accuracy by 5.7% while reducing model parameters by 34% compared to the baseline.
- Successfully integrated LAMP pruning and knowledge distillation techniques to create a lightweight vehicle detection model with only 14.1 GFLOPs computational cost.

**What is the implication of the main finding?**

- The proposed approach effectively addresses vehicle detection challenges in complex urban traffic scenes, significantly reducing false positives and missed detections.
- The lightweight yet accurate model design enables practical deployment in resource-constrained environments while maintaining high detection performance.

**Abstract:**

Aiming at the problems of insufficient vehicle detection accuracy, high misdetection and omission rate, and heavy model computational burden caused by complex lighting conditions, target occlusion, and other factors in urban traffic scenarios, this paper proposes an improved lightweight detection network, ZZ-YOLO. Firstly, the current mainstream target detection algorithms lack components to improve the network’s focus on the edges of the objects, which can indirectly lead to unclear classification and localization. For this reason, in this paper, we self-develop a module of GlobalEdgeInformationTransfer (GEIT), which can help us to transfer the edge information extracted from the shallow features to the whole network and fuse it with the features of different scales. Secondly, to reduce the number of parameters in the detection head and to fuse the extracted features better, a self-developed Lightweight Detail Convolutional Detection Head (LDCD) detection head is introduced. After that, the most effective layer-adaptive magnitude-based pruning (LAMP) method is used to build away the redundant parameters to make the detection network more lightweight. Finally, in order to ensure that the detection accuracy of the pruned model will not be too low, a model distillation method was used, in which YOLOv11x + LDCD was used as the teacher model and the pruned model was distilled as the student model. Experimental data on the optimized KITTI and BDD100K datasets show that the detection accuracy of the ZZ-YOLO algorithm is 70.9%, the mAP (mean Average Precision) @0.5 is 58%, the model-parameter quantity is 14.1GFLOPs compared to the original algorithm, the detection accuracy is increased by 5.7%, and the average precision is increased by 2.3%. The amount of model parameters is reduced by 34%, and the real-vehicle verification session effectively reduces the misdetection and omission of vehicles.

## 1. Introduction

With the booming development of artificial intelligence technology and the in-depth promotion of smart city construction, the intelligent transportation system, an important part of urban infrastructure, has put higher requirements for real-time and accurate target detection technology. Target detection technology in road scenarios not only needs to accurately identify and locate key targets such as vehicles, pedestrians, traffic signs, etc., but also needs to deal with complex and changing environmental factors to provide reliable data support for intelligent applications such as traffic flow analysis, accident early warning, and automatic driving. However, in practical application scenarios, especially in adverse weather conditions, complex lighting environments, and when facing high-speed moving objects and small targets at long distances, existing detection algorithms often find it difficult to maintain stable performance, resulting in a serious impact on the reliability and practicality of detection results [1].

The rise of deep learning has revolutionized the field of target detection. Detection algorithms based on deep neural networks can be broadly categorized into two major groups: two-stage detection algorithms and one-stage detection algorithms. The R-CNN family, including R-CNN, Fast R-CNN, Faster R-CNN, etc., represents two-stage detection algorithms. Cai et al. [2] proposed the Cascade R-CNN framework, which gradually improves the detection frames by designing a multi-stage detector cascade structure with progressive IoU thresholds, where the outputs from the previous stage of the detector at each stage serve as the training samples and gradually improve the detection frame quality, improving the AP to 42.8% on the COCO dataset, which is 2.3 percentage points higher than the benchmark Faster R-CNN. Lin et al. [3] designed a feature pyramid network (FPN), innovatively constructed top-down feature-enhancement paths, and established lateral connections between feature maps with different resolutions, realizing the effective fusion of multiscale features, which makes the detector obtain rich semantic information and spatial details when dealing with targets at different scales. Dai et al. [4] proposed Deformable Convolutional Networks (Deformable ConvNets), which significantly enhanced the model’s ability to model target deformation by learning additional spatial offsets so that the convolution kernel can dynamically adjust the sampling position according to the shape of the target. He et al. [5] proposed a top-down feature augmentation path based on the Faster R-CNN and established horizontal connections between different resolution feature maps, which realized the effective fusion of multiscale features, enabling the detector to obtain rich semantic information and spatial details when dealing with targets of different scales. Adding the instance segmentation branch to Faster R-CNN proposed the Mask R-CNN framework, which guarantees the accuracy of feature alignment through the RoIAlign layer and achieves 43.3% box AP and 37.2% mask AP on the COCO dataset.

The YOLO series has made significant progress in vehicle detection as representative of first-stage detection algorithms.

The YOLOv4 designed by Bochkovskiy et al. [6] contains the following key improvements: (1) proposing the Cross-Stage Partial Connection (CSP) module, which optimizes the computational efficiency and accuracy; (2) using the Bag of Freebies and Bag of Specials methods to enhance the performance of target detection; and (3) introducing the Mish activation function and the optimized IoU loss function are introduced to particularly enhance the detection of small targets. An AP of 43.5% is achieved on the MS COCO dataset, which is especially good in real-time detection tasks. However, in complex traffic environments, occlusion and light variations still lead to the degradation of detection accuracy and problems of false and missed detections.

YOLOv3, designed by Redmon et al. [7], contains the following key improvements: (1) proposing a multiscale prediction method to significantly improve the performance of small-target detection; (2) using Darknet-53 as the backbone network, combined with the residual network structure to enhance the feature extraction capability; and (3) introducing a logistic loss function to improve the classification performance and bounding box prediction accuracy. An AP of 33.0% is achieved on the PASCAL VOC and MS COCO datasets, particularly suitable for small-target vehicle detection in complex scenes. However, occlusion and light changes still have some impact on the stability of the algorithm, which can easily lead to the phenomenon of false or missed detection.

The YOLO-based T-YOLO model proposed by Carrasco et al. [8] contains the following key improvements: (1) proposed an improved model T-YOLO based on YOLO and multiscale convolutional neural network (MSCNN) for optimizing the performance of small vehicle detection; (2) enhanced the detection capability of small target vehicles by introducing a multiscale feature extraction module while maintaining the real-time detection high efficiency; and (3) achieved 40.7% and 38.2% mAP on VisDrone and UAVDT datasets, respectively, and the inference speed on embedded devices is significantly better than the traditional YOLO model. However, there are still some false detection problems in target-intensive scenarios.

The Fast-Yolo-Rec model, based on the YOLO and recurrent network proposed by Zarei et al. [9], contains the following key improvements: (1) a model Fast-Yolo-Rec combining YOLO detection network and recurrent neural network (RNN) is proposed for accelerating vehicle detection in consecutive video frames; (2) YOLO is used for the initial detection, and a recurrent prediction network is utilized to predict the target position in successive frames, thus reducing repeated computation and improving processing speed; (3) achieved 95.1% detection accuracy on the UA-DETRAC dataset, while real-time performance of 30 FPS on successive frames was realized. However, the model’s prediction accuracy decreases when detecting fast-moving targets.

Miao et al. [10] proposed a YOLOv3-based nighttime vehicle detection method that (1) proposes an improved YOLOv3-based model focusing on nighttime vehicle detection and enhances the detection of targets under low-light conditions by improving the feature extraction module; (2) introduces image preprocessing techniques (e.g., histogram equalization) to optimize the quality of the input image and adopts a multiscale detection strategy to improve the detection of vehicles of different sizes; (3) achieves 91.5% mAP on a self-constructed nighttime traffic dataset, which is significantly better than the traditional detection algorithms. However, there is still room for improvement in the false detection rate in scenes with strong light interference (e.g., headlight glare).

Zhu et al. [11] proposed a MME-YOLO model based on multisensor multilayer enhancement: (1) A multisensor multilayer enhancement YOLO model (MME-YOLO) is proposed to combine multimodal data (e.g., RGB images and infrared images) for vehicle detection in order to enhance robustness under complex traffic environments; (2) a multisensor multilayer enhancement YOLO model is proposed to effectively combine different scales features through the introduction of a Multi-Level Feature Fusion, which effectively combines different scale features to enhance the detection performance in small target detection and occlusion cases; (3) it achieved 93.2% and 92.7% mAP on M3D and KITTI datasets, respectively, which shows strong adaptability to complex environments (e.g., illumination changes, occlusion, etc.). However, the computational overhead associated with multisensor data fusion limits its real-time performance.

Song et al. [12] proposed a vehicle detection method based on MEB-YOLO (1) an efficient vehicle detection model, MEB-YOLO, which enhances the feature-extraction capability by introducing Mixed Enhancement Block (MEB) in complex traffic scenarios; (2) employed an improved Feature Pyramid Network (FPN) structure for multiscale feature fusion, which enhances the detection performance of small-target vehicles and partially occluded targets; and (3) achieved 94.6% and 92.3% mAP on the KITTI and BDD100K datasets, respectively, while maintaining a high real-time performance (38 FPS). However, the detection accuracy remains degraded under extreme lighting conditions (e.g., strong reflections or very dark environments).

Huang et al. [13] proposed a nighttime vehicle detection method based on M-YOLO. (1) A lightweight vehicle detection model M-YOLO combining MobileNet v2 and YOLOv3 is proposed, which reduces the computational complexity by adopting MobileNet v2 as the backbone network and is suitable for both embedded and real-time applications; (2) for the nighttime low-light condition, M-YOLO introduces a feature enhancement module and image preprocessing steps (e.g., contrast enhancement and noise removal), which improves the detection accuracy of nighttime vehicles; (3) 89.7% mAP is achieved on a self-constructed nighttime traffic dataset, and the inference speed reaches 42 FPS, demonstrating good real-time performance. However, the model still suffers from certain false detection and leakage rates under strong light interference (e.g., headlight glare) scenarios.

When vehicle detection is performed in complex traffic environment scenarios, problems such as light changes, occlusion, and target density still pose significant challenges to the performance of detection algorithms. Although these algorithms show high detection accuracy and real-time performance in specific scenarios, they still suffer from misdetection and omission problems to varying degrees.

To address the above problems, this paper proposes an improved lightweight detection network, ZZ-YOLO, which mainly contains the following improvement points:

(1) The GlobalEdgeInformationTransfer (GEIT) module is designed to improve the network’s focus on object edges, effectively enhancing the clarity of target classification and localization. This module can transfer the edge information extracted from shallow features to the whole network and fuse it with features of different scales.
(2) A lightweight LDCD detection head is proposed to optimize the feature fusion effect while reducing the number of parameters.
(3) The LAMP pruning method optimizes the network, reducing the model’s computational complexity.
(4) The model distillation technique is introduced, and YOLOv11x + LDCD is used as a teacher model to guide the learning of the lightweight model after pruning, ensuring that the model performance will not be significantly degraded by pruning.

## 2. Materials and Methods

### 2.1. YOLO11 Introduction

YOLOv11 is a new generation of target detection model developed by Ultralytics as an advanced version of YOLOv8, which realizes significant improvements in several aspects.

First, regarding network architecture, YOLOv11 has made innovative changes to the backbone network (backbone) by replacing the C2f module in YOLOv8 with the better-performing C3k2 structure.

Second, YOLOv11 introduces an innovative C2PSA feature-enhancement module. This module combines the efficiency of the C2 structure with the advantages of the Position Self-Attention mechanism, which significantly improves the model’s ability to capture target position information and spatial dependencies, resulting in more accurate target detection.

Then, on top of the original decoupled detector head design, YOLOv11 optimizes the classification detector head by adding two depth-separable convolutional layers (DWConv). This dramatically reduces the number of parameters and the computational overhead of the model while maintaining its performance, making it more suitable for practical deployment applications.

**Figure 1:**
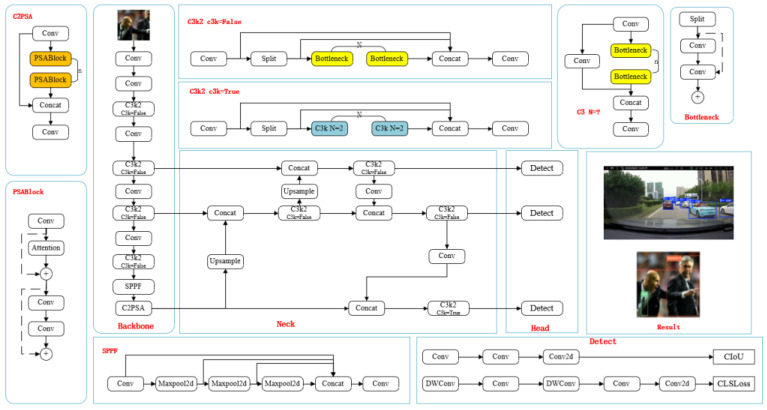
YOLOv11 network structure diagram.

Finally, YOLOv11 moderately increases the depth and width of each version of the network model while maintaining high efficiency, and this structural expansion further enhances the feature extraction capability of the model to deal with more complex detection scenarios. The network structure of YOLOv11 is shown in Figure 1 below. Since the original YOLOv11 algorithm cannot satisfy the current detection in traffic scenarios due to the mutual occlusion of vehicles and the influence of light, YOLOv11 is used as a baseline model for improvement [14].

### 2.2. Improvement of YOLOv11 Network

#### 2.2.1. Global Edge Generation Transformation Module (GEIT)

**Figure 2:**
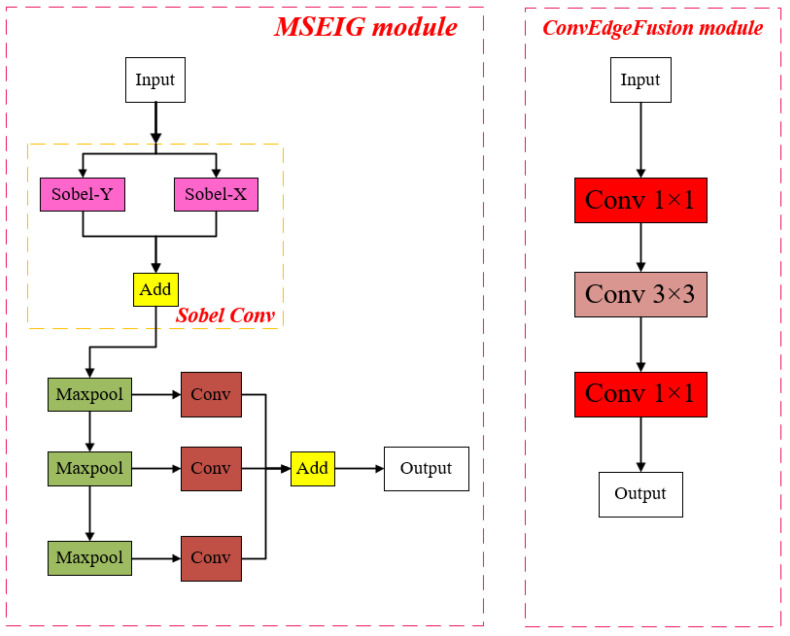
MSEIG module and ConvEdgeFusion module internal diagrams.

The localization of the object frame is very much dependent on the edge information of the object. However, for conventional target detection networks such as YOLOv8, YOLOv9, and RT-DERT, no component can improve the network’s focus on the object’s edge information. A module needs to be developed to allow the edge information to be fused with the features extracted from various scales. Hence, the paper proposes a GlobalEdgeInformationTransfer (GEIT) module, which can transfer the edge information extracted from shallow features to the whole network and fuse it with features at different scales. Its structure contains two parts: a multiscale edge information guidance module (MSEIG Module) and an edge information fusion module (ConvEdgeFusion Module), and its structure is shown in Figure 2 below.

##### Introduction to the Global Edge Generation Conversion Module

Multiscale Edge Information Guidance Module (MSEIG)

Since the original image usually contains a large amount of redundant background information, if the edge information of the original image is directly extracted and passed to the whole network, significant noise interference will be introduced, thus affecting the convergence of the model. Considering that the shallow convolutional layer itself has the property of filtering irrelevant background information, the paper designs a multi-scale edge information generation module (MSEIG) embedded in the shallow layer of the network. The module innovatively utilizes the shallow feature layer to generate multi-scale edge feature maps and injects them into different layers of the backbone network through a cross-scale fusion mechanism.

The core component of the MSEIG module is an edge detection structure based on the Sobel operator, where the Sobel-X operator is dedicated to extracting the spatial gradient features in the horizontal axis (x-axis) of the image, while the Sobel-Y operator is responsible for capturing the feature changes in the vertical axis (y-axis). This dual-channel edge detection mechanism can effectively construct multi-scale edge representations and provide more discriminative geometric structure information for the deep network.

Optimization of the Downsampling Strategy

In edge information processing, the choice of downsampling strategy is crucial. In order to retain and enhance the edge information while achieving effective downsampling, MaxPool is chosen after experimental validation, which can retain the salient features in the local area, effectively highlight the edge information, and avoid the edge intensity being diluted or blurred in the downsampling process. In contrast, average pooling (AvgPool), although suitable for scenes requiring smoothing or homogenizing features, performs significantly worse than MaxPool in preserving fine edge details, especially when dealing with high-contrast boundaries.

Convolutional Edge Fusion Mechanisms

In order to realize the efficient integration of edge information and regular convolutional features, the Convolutional Edge Fusion (ConvEdgeFusion) module is designed. The module adopts a three-stage processing strategy. Firstly, the cross-channel fusion of edge information and regular convolutional features is realized by conv_channel_fusion (1 × 1 convolution), so that the features from two different sources can fully interact with each other in the channel dimension, and the correlation between the features is established. Second, conv_3 × 3_feature_extract is applied for feature extraction and spatial context integration to enhance the model’s ability to capture local structural patterns, which is crucial for accurately characterizing edge and texture details. Finally, feature space remapping is performed via conv_1 × 1 to adjust the dimensional composition of the output features so that they can be seamlessly integrated into the subsequent processing stages of the network.

**Figure 3:**
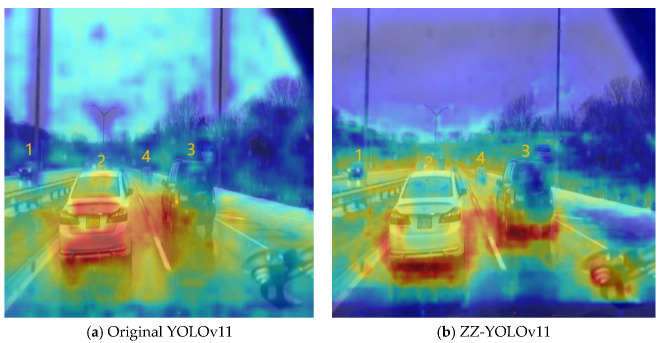
Heat map of attentional focus in marginal regions.

In order to verify the feasibility of adding edge modules, an edge heat map (shown in Figure 3 below) was used to illustrate the feasibility of edge modules. As can be seen in the figure, in serial numbers 1 and 4, it can be seen that using the edge module, its warm color is more gathered around the vehicle, while not using the edge module is surrounded by cold color. In serial numbers 2 and 3, it can be seen that the warm colors of the edge module are more gathered around the vehicle, while the warm colors of the unused edge module are more scattered. It can be seen that the use of the edge module can be more conducive to the localization of the vehicle.

#### 2.2.2. Lightweight LDCD Detection Head

**Figure 4:**
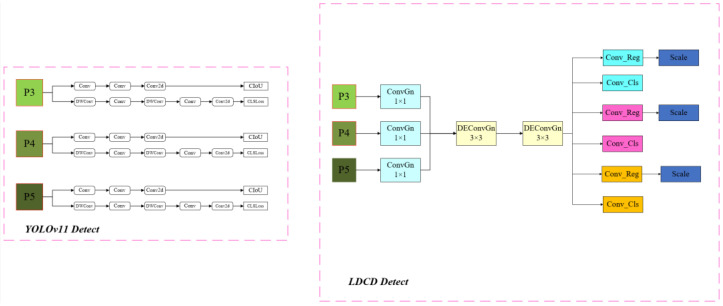
Exploded view of the internal structure of the LDCD detector head.

Although YOLOv11 incorporates the addition of two depth-separable convolutional layers (DWConv) in the detection head part, which dramatically reduces the number of parameters and computational overhead of the model while maintaining the performance, the number of parameters in its detection head is still at 30% of the network as a whole. In order to make the model lighter, the improvement of the detection head is indispensable; for this reason, the paper redesigned the head, and the new LDCD detection head structure is shown in Figure 4 below.

##### Characteristics of the New Inspection Head

Strategic Update of the Normalization Layer

To cope with the training challenges posed by the increasing size of deep-learning models, the paper uniformly replaces the normalization layer in the network with Group Normalization (GN) [15]. As the model parameter size increases, traditional normalization methods are limited in small batch training scenarios when the batch size shrinks due to computational resource constraints, the model performance degrades significantly. GN effectively circumvents the dependence on the batch size by grouping the normalization on the channel dimensions and ensures stable training results under the restricted batch size conditions. The experimental results echo the FCOS [16] study, confirming that GN significantly improves the performance of detection networks on target localization and classification tasks.

Lightweight Design of the Detection Head

Traditional detection head architectures usually require three separate feature-extraction operations for classification and localization tasks, leading to a high parameter count and computational complexity, and it is unsuitable for resource-constrained deployment environments. To address this issue, the paper proposes a lightweight detection head design based on parameter sharing, combining the three separate feature extraction operations into a single operation, which is subsequently scaled by a dedicated scale module. This improvement significantly reduces the computational burden of the model while maintaining detection performance, making the network more suitable for real-time deployment on devices with limited computational resources.

Introduction of Detail-Enhanced Convolution Module

**Figure 5:**
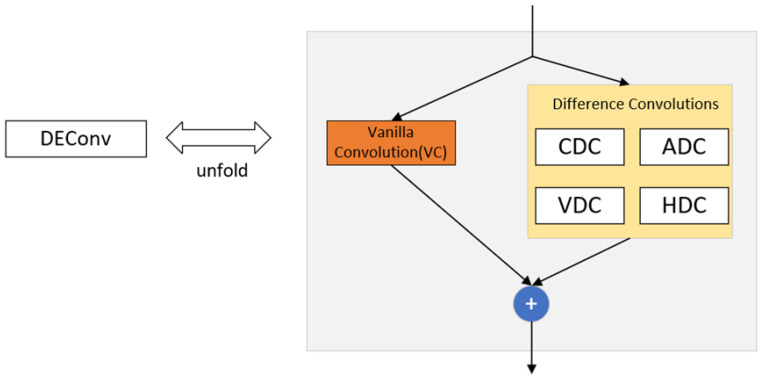
Detailed-enhanced convolutional architecture diagram.

Detail-enhanced convolution (DEConv) [17] is a special convolutional structure designed for enhanced detail perception, whose structure is shown in Figure 5 below. DEConv contains five convolutional layers (four difference convolution kernels and one normal convolution) that are deployed in parallel for feature extraction. Specifically, central difference convolution (CDC), angular difference convolution (ADC), horizontal difference convolution (HDC), and vertical difference convolution (VDC) are used to integrate traditional local descriptors into the convolutional layers, which allows for enhanced representation and generalization capabilities. Their main features are as follows:

(1) Dual-path structure: DEConv adopts a parallel dual-path design, where one path handles the main semantic information, and the other focuses on detailed feature extraction.
(2) Adaptive Feature Enhancement: Dynamically adjusts the importance of detail information through a feature-reweighting mechanism, enabling the network to intelligently focus on key detail regions based on the input content.
(3) Multiscale receptive fields: Integrate different scales of receptive fields to simultaneously capture local fine structure and global context information.
(4) Edge retention capability: Specially optimized to retain high-frequency information such as edges and textures, reducing the loss of detail common in traditional convolutions.
(5) Pluggable design: Standard convolutional layers in an existing network can be directly replaced without drastically changing the network architecture.

#### 2.2.3. Model Pruning

In order to further reduce the number of parameters and computation of the model, the paper uses the most proven LAMP pruning method [18], which is characterized as follows:

##### Hierarchical Adaptive Analysis

LAMP first performs a fine-grained layer assessment to analyze how each network layer affects the final performance. A complete layer-sensitivity map is constructed by integrating multi-dimensional metrics, such as weight distribution, gradient change, activation statistics, and information flow. This analysis identifies which layers contain redundant information and which layers undertake critical computations, providing a scientific basis for pinpointing pruning targets.

##### Multi-Granularity Pruning Strategies

Based on the sensitivity analysis, LAMP selects the optimal pruning granularity for layers with different characteristics. Conservative channel pruning may be applied to the early convolutional feature extraction layers; structured pruning may be applied to the computationally intensive intermediate layers; and deep-weight sparsification may be implemented to the highly redundant, fully connected layers. This differentiated treatment ensures that the pruning scheme matches the functional characteristics of each part of the network.

##### Progressive Pruning and Monitoring

LAMP uses a progressive iterative pruning process to decompose the compression task into multiple small steps. After each round of pruning, the system evaluates the changes in model performance and feeds the results back to the next round of decision-making. This real-time monitoring and dynamic adjustment mechanism allows for the early detection of potential problems, prevents excessive pruning leading to a sharp drop in performance, and ensures a smooth and controllable compression process.

Network Restructuring and Optimization

After completing parameter pruning, LAMP performs structural reorganization to optimize the computational flow of the pruned network. This includes reorganizing computational units, merging functionally similar operations, adjusting data-flow direction, and optimizing memory access patterns. The restructuring eliminates the fragmentation problem introduced by pruning and improves the model’s execution efficiency on the target hardware.

**Figure 6:**
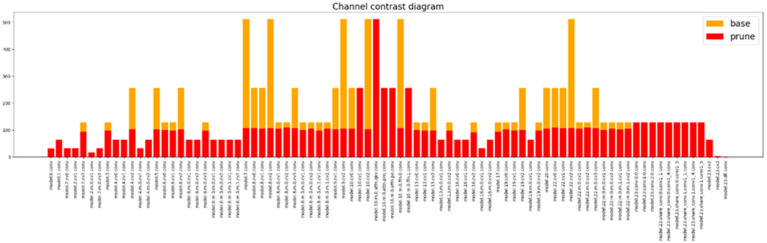
Comparison plot of modeled pruning channels.

Figure 6 below compares the pruned channels, where the base is the unpruned model and the prune is the pruned model.

#### 2.2.4. Knowledge Distillation

To address the problem of the loss of detection model accuracy due to pruning operations, this study adopts the knowledge-distillation technique as an effective means of compensation. Knowledge distillation is a model-capability migration mechanism that extracts key knowledge from complex models with rich parameters and excellent performance and effectively transfers this knowledge to lightweight models with significantly reduced parameters. This process can be viewed as the transfer of expertise from a network of “knowledgeable” teachers to a network of “lightweight” students, enabling small networks to exhibit performance close to that of large models in resource-constrained environments [19].

**Figure 7:**
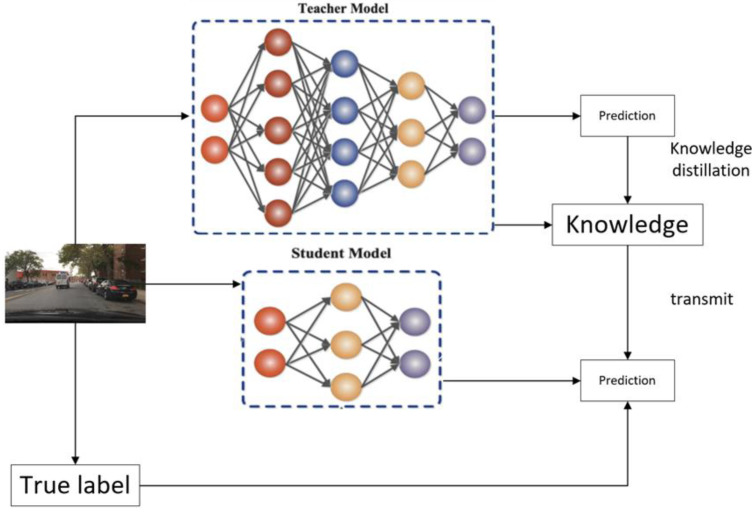
Schematic diagram of knowledge distillation.

The knowledge-distillation framework is based on the teacher–student model, whose schematic diagram is shown in Figure 7, and its execution process consists of the following stages:

First, a high-capacity teacher model (Net-T) is constructed and trained. The model is usually complex in structure, rich in parameters, or integrated by multiple expert models. The basic function of the teacher model is to generate softmax-processed probability distribution outputs for the input data X. These outputs contain rich category similarity information.

Next, a lightweight student model (Net-S) is designed and trained. This model has a streamlined structure and fewer parameters, but it maintains the same input–output interface specification as the teacher model. The student model also needs to generate softmax probability distributions for input X, but it may not perform as well as the teacher model initially.

On this basis, the teacher model transfers its captured knowledge of data features and decision boundaries to the student model under its powerful learning ability and rich expressive capability. This knowledge transfer significantly enhances the student model’s ability to generalize to unseen data, enabling it to approximate the performance level of the teacher model while maintaining its lightweight advantage [20].

**Figure 8:**
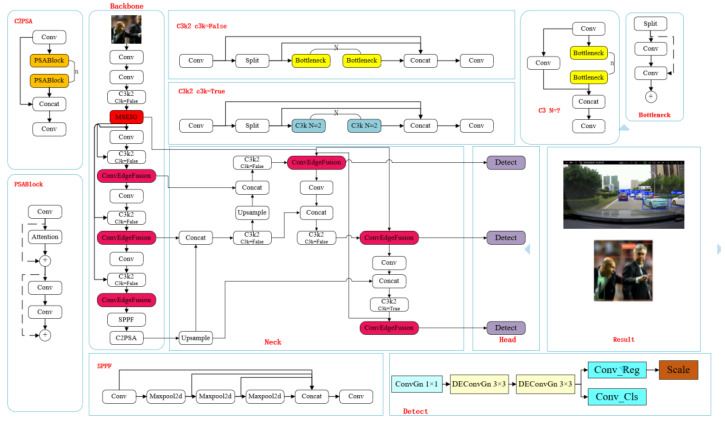
ZZ-YOLOv11 network structure diagram.

The following improvements are made in the YOLOv11 network: First, the edge information guidance module (MSEIG) is placed in front of each detection layer of the YOLOv11 backbone network to extract features and use them for the subsequent integration of information in the feature pyramid. This approach enables the edge information to be better delivered to each detection layer, so that each detection layer can utilize the edge information to improve the detection efficiency, as shown by the dark-red module in Figure 8. Secondly, in order to allow the subsequent feature splicing to utilize the edge information, the edge information module (ConvEdgeFusion) is placed in front of the Concat module, and its specific location is shown in the dark-red module in Figure 8. Finally, the original detector head is replaced by the self-developed LDCD detector head, and the position of the replaced head is shown in the purple module in Figure 8.

## 3. Experimental Design and Validation

### 3.1. Selection of Dataset and Experimental Environment

The training dataset used in the paper is from a portion of the publicly available KITTI and BDD100K datasets, which contains a total of 15,000 sheets of nighttime scenes, vehicle-to-vehicle mutual occlusion, and obstacle occlusion of vehicles and normal scenes, and is divided into training, test, and validation sets by 7:3:1. The computer configuration used is: Linux operating system, 16 vCPU Intel (R) Xeon (R) Platinum 8474C, RTX 4090D GPU, 24 GB RAM (Lenovo, produced in Hong Kong, China), and the environment used is Pytorch 1.13.0, Cuda 11.7, and Python 3.8. For the real-vehicle validation session, the smart vehicle data-acquisition platform of the in-vehicle smart camera is used to realize it.

### 3.2. Evaluation Indicators for the Experiment

The accuracy and real-time performance of the algorithm’s detection are important indicators for evaluating the algorithm’s application to real scenarios. Therefore, this paper selects the evaluation metrics for accuracy and real-time performance. These metrics include detection accuracy (P), mean accuracy (mAP), the number of parameters of the model (Pa), and the computation of the model.

Equation (1) shows the formula for detection accuracy (P). It represents the proportion of positive samples correctly recognized by the model over all samples, and a higher detection accuracy represents a higher probability of target recognition by the model [21].

(1) $$P=\frac{TP}{TP+FP}$$

mAP indicators are divided into mAP@0.5 and mAP@0.5–0.95. mAP@0.5 represents the average accuracy when the IoU threshold is 0.5, and mAP@0.5–0.95 represents the average accuracy on different IoU thresholds (from 0.5 to 0.95, step size 0.05). IoU is an Intersection over Union, which measures the degree of overlap between the prediction box and the real box. The specific content is shown in Formula (3):

(2) $$AP=\int_{0}^{1}PR\mathrm{dr}$$

(3) $$mAP=\frac{1}{c}\sum_{j=1}^{c}AP_{i}$$

Parameter count (Pa) is the total number of parameters in the model during training, including weights, bias values, etc. The model parametric quantity is used to measure the spatial complexity of the model and the model; a larger parametric quantity means a higher complexity of the model and higher memory required.

Model computations (GFLOPs) are the number of floating-point operations the model performs in 1 s. They are used to evaluate the model’s complexity and computational efficiency.

### 3.3. Ablation Test

In order to systematically evaluate the impact of the Global Edge Generation Transformation Module (GEIT), Lightweight Detection Header (LDCD), model pruning, and knowledge refinement techniques on the performance of the YOLOv11 algorithm, a series of well-designed ablation experiments were conducted in this study. YOLOv11s was used as the baseline model. Each set of experiments was conducted for 200 full training rounds, and the experimental results are detailed in Table 1.

Data analysis shows that the introduction of the LDCD detection head successfully reduces the number of model parameters and computational burden while improving the detection accuracy and average accuracy. Meanwhile, the GEIT module significantly improves the detection accuracy and drastically reduces the consumption of computational resources. Compared with the original YOLOv11s, the combination of the YOLOv11s-GEIT-LDCD scheme achieves an excellent performance balance and significantly improves the accuracy. The combination of the YOLOv11s-GEIT-LDCD scheme achieves an excellent performance balance and significantly improves the accuracy compared to the original YOLOv11s while effectively alleviating the resource pressure brought about by using GEIT alone. At the same time, it effectively alleviates the resource pressure brought by the use of GEIT alone.

On this basis, the application of the model pruning technique greatly reduces the computational burden, but it also results in a significant decrease in the detection performance. In order to solve this challenge, the paper introduces a knowledge-distillation mechanism, which successfully recovers most of the detection accuracy lost due to pruning and realizes the optimal trade-off between performance and efficiency without adding any additional resource consumption, where YOLOv11x-LDCD is used as the teacher model for distillation, and YOLOv11s- GEIT-LDCD-Prune is the result after pruning and used as the student model for knowledge distillation.

**Figure 9:**
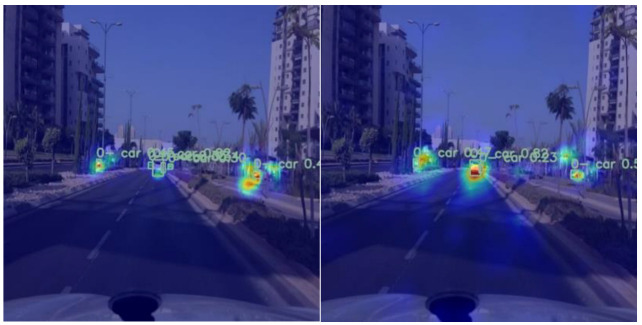
HiResCAM–heatmap distribution of YOLOv11s vs. ZZ-YOLOv11.

To further illustrate the performance of the improved model, the HiResCAM-heatmap [22] was used, and the results are shown in Figure 9 below ZZ-YOLOv11, demonstrating a more focused and accurate heatmap distribution. The heat region in the right panel is more focused on the actual vehicle location, and the change in heat intensity is smoother and more natural, indicating that the algorithm is more deterministic about the target location. In contrast, YOLOv11 heat distribution is more dispersed, and the boundaries are unclear.

### 3.4. Comparative Experiment

In order to evaluate the effectiveness of the method proposed in this paper, the improved method was compared with Faster-RCNN [23], SSD [24], RT-DERT [21], RT-DERT-r50, YOLOv5, YOLOv6 [25], YOLOv8 [18], YOLOv9 [26], and YOLOv10 [27] in the table on the same experimental dataset [28]. The experimental results are shown in Table 2.

The experimental results show that the improved algorithm outperforms the current target detection algorithm on the public dataset in terms of detection accuracy, average accuracy, number of parameters, computation, and memory. The improved algorithm is more suitable for the detection of vehicles than the current mainstream detection algorithms and for the deployment and application of the detection model to small devices.

### 3.5. Generalization Test

**Figure 10:**
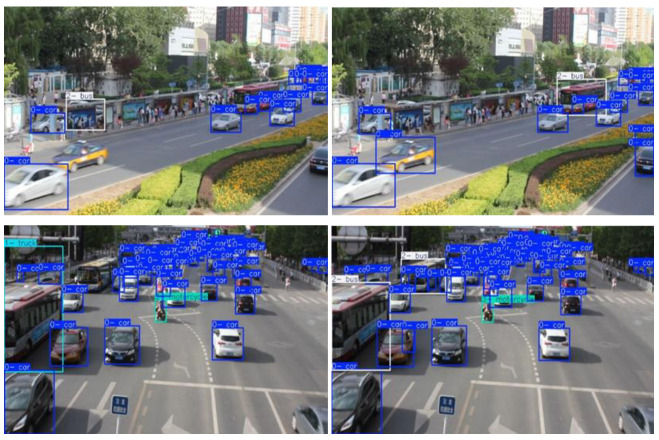
Comparison chart of generalization test.

The UA-DETRAC-G2 public dataset was selected to verify the improved algorithm’s effectiveness and reasonableness on other public datasets. The results are shown in Figure 10 below. The left side shows the results detected by the original YOLOv11s algorithm, and the right side shows the results detected by the improved ZZ-YOLOv11 algorithm.

In order to comprehensively evaluate the performance of the algorithm, the paper selects two high-complexity (e.g., high-traffic and shaded) traffic scenarios for comparative testing. In the first high-traffic environment, the ZZ-YOLOv11 algorithm shows obvious advantages, with significantly lower false and missed detection rates than the original YOLOv11s algorithm, successfully capturing more vehicle targets while effectively reducing false recognition.

The second test scenario was chosen at a cross-traffic intersection with a higher complexity of vehicle intersections, with a special focus on the detection performance of oncoming vehicles in the opposite direction. In this challenging dynamic environment, the ZZ-YOLOv11 algorithm also maintains excellent detection stability, which not only reduces the number of missed detections but also significantly reduces the frequency of false detections compared to the original YOLOv11s model, ensuring the reliability of the detection results.

The comparative experimental results of these two sets of typical traffic scenarios strongly demonstrate the feasibility and generalization ability of the ZZ-YOLOv11 algorithm in practical application environments. The algorithm shows consistent performance advantages under different traffic conditions, provides more reliable technical support for intelligent vision systems in complex road environments, and further verifies the effectiveness of the improvement strategy proposed in the paper in practical applications.

### 3.6. Real Vehicle Tests

**Figure 11:**
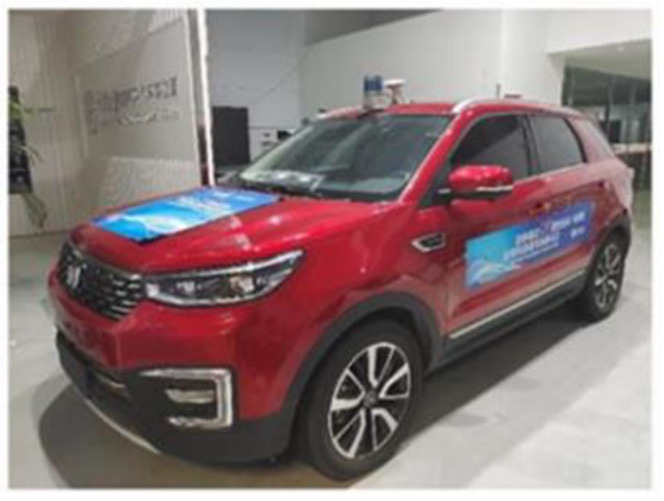
Data-collection intelligent vehicle full view.

The paper conducts a real-vehicle test to verify the algorithm’s feasibility in real scenes. The test is based on the intelligent vehicle data-acquisition platform, which acquires the real traffic scene data through the onboard intelligent camera. The data-acquisition vehicle is shown in Figure 11.

**Figure 12:**
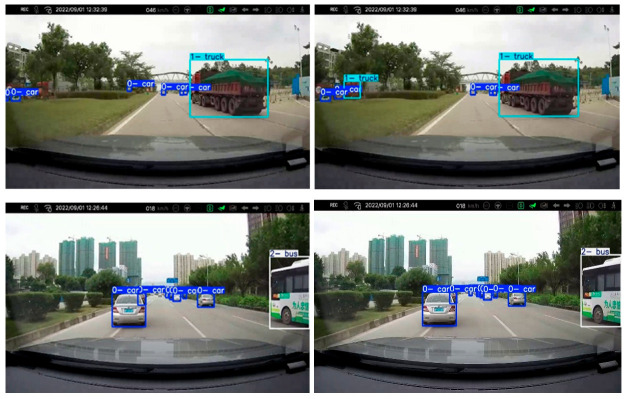
Comparison chart of the effect of real vehicle testing.

Figure 12 compares the actual detection capabilities of the original YOLOv11s and the improved ZZ-YOLOv11. In the first test scenario, faced with a truck target partially obscured by leaves, the original YOLOv11s algorithm completely fails to recognize the target, while the improved ZZ-YOLOv11 algorithm successfully captures and accurately identifies the obscured object.

Similarly, in the second scenario test, the YOLOv11s algorithm also exhibits a detection blind spot and fails to recognize distant vehicle targets when the target is a distant front vehicle. In contrast, the ZZ-YOLOv11 algorithm demonstrated significantly enhanced long-range detection capabilities, successfully marking these critical targets.

These two sets of comparison experiments vividly demonstrate the excellent performance of the ZZ-YOLOv11 algorithm in dealing with the challenges of real-world applications, such as occluded object detection and long-distance target recognition, and they fully validate the practicability and reliability of the improved algorithm in real complex environments. These advantages significantly improve safety and effectiveness in key application scenarios such as autonomous driving and intelligent surveillance.

## 4. Conclusions

This study proposes the ZZ-YOLOv11 algorithm based on YOLOv11 to explore a vehicle recognition method that balances lightweight and detection accuracy, with the following main improvements:

Global Edge Information Transfer (GEIT) module extracts edge features from the shallow network and transfers them to the deep layer, which promotes multi-scale feature fusion and target contour perception, and it improves classification and localization performance.

Lightweight Depth Detection Head (LDCD) provides ideas for lightweight model design by optimizing the feature fusion architecture to reduce the number of parameters while maintaining fusion efficiency.

The LAMP pruning method is applied to remove redundant connections and parameters, reduce model complexity, and adapt to resource-constrained environments.

A knowledge-distillation framework is constructed with YOLOv11x + LDCD as the teacher model to compensate for the performance loss caused by pruning.

On the KITTI and BDD100K datasets, the detection accuracy of the ZZ-YOLO algorithm is about 70.9%, mAP@0.5 is 58%, and the computation amount is 14.1GFLOPs, which improves the accuracy and reduces the parameter amount compared with the basic algorithm. The real vehicle test shows that the algorithm can reduce the vehicle misdetection and omission rate.

Despite the progress, the algorithm still has room for improvement in detection accuracy. Future research will be devoted to further improving detection accuracy while maintaining lightweight, exploring more efficient feature extraction methods and model structure optimization schemes, and providing more practical technical references for vehicle detection.

## Figures and Tables

**Table 1:** Ablation trial.

Model	P/%	mAP0.5/%	mAP0.5–0.95/%	Participant Number	Floating-Point Calculations/GFLOPs
YOLOv11s	65.2	55.7	37.5	9,414,348	21.3
YOLOv11s-LDCD	65.6	57.6	38.1	8,915,751	19.8
YOLOv11s-GEIT	76.2	57	38.3	12,721,804	33.3
YOLOv11s-GEIT-LDCD	69.8	57.4	37.8	11,963,527	30.2
YOLOv11s- GEIT-LDCD-Prune	66.3	56.7	38.2	2,449,743	14.1
YOLOv11x	75.8	61	41.1	56,831,644	198.4
YOLOv11x-LDCD	76.9	61.6	41.7	54,109,719	193.2
YOLOv11s-All	70.9	58	39.2	2,449,743	14.1

**Table 2:** Comparison test.

Model	P/%	mAP0.5/%	mAP0.5–0.95/%	Participant Number	Floating-Point Calculations/GFLOPs
F-RCNN	50.1	46.4	28.5	284,780,223	186.9
SSD	56.3	49.5	30.3	25,934,879	61.9
RT-DERT-X	58.8	48.7	31.8	28,489,674	101.2
RT-DERT-r50	60.2	50.5	32.6	41,897,973	126.1
YOLOv5s	58.4	49.7	32.3	7,815,164	18.7
YOLOv6s	59.7	49.8	33.1	15,976,924	42.8
YOLOv7	64.2	54.1	36.8	29,087,126	102.2
YOLOv8s	64.8	52.1	34.3	9,829,212	23.4
YOLOv9s	62.3	53.7	35.2	6,195,196	22.1
YOLOv10s	58.1	49.4	32.3	7,219,548	21.4
YOLOv11s	65.2	55.7	37.5	9,414,348	21.3
ours	70.9	58	39.2	2,449,743	14.1

## Data Availability

The data used to support the findings of this study are available from the corresponding author upon request.
